# Benzoate of Sodium in Uremia

**Published:** 1888-04

**Authors:** 


### *BENZOATE OF SODIUM IN UREMIA.*

Parzevski puts forth some remarkable claims regarding the curative power of sodium benzoate in uremic intoxication (quoted in the *Bull. Gen. de Therap.*, Dec. 15, 1887). He administers four to eight grammes (ʒj-ʒij) a day, in solution or capsule, the latter preferably. Twice the quantity can be given by the rectum, if well diluted. Under the action of this remedy, the paroxysms lessen in severity, the intervals grow longer, and the convulsions, after a time, cease entirely. Profound sleep is produced by it, and during this the cerebral functions are restored. When albuminuria exists, a marked diminution occurs in the quantity present, or the albumen disappears entirely.

[If the claims are substantiated, they will furnish another strong argument in favor of the benzoate of soda in scarlet fever and diphtheria. Why it should be given in capsules, does not appear.

On general principles, all remedies are better when given in solution; and when they are so far removed from being disagreeable as is this drug, the liquid form is certainly to be preferred. The following formula is an agreeable way of administering it:

| | | |
|---|---|---|
| ℞ | Benzoate soda | ℈iv. |
| | Glycerine | ℥ss. |
| | Aquæ menth. p. | ℥iss. |

M. Sig.—Teaspoonful every hour or two for an infant.]—*Weekly Med. Review.*

